# Efficacy and safety of dual-task training in enhancing cognitive and motor recovery for post-stroke rehabilitation

**DOI:** 10.1097/MS9.0000000000004458

**Published:** 2025-11-27

**Authors:** Nicholas Aderinto, Israel Charles Abraham, Gbolahan Olatunji, Emmanuel Kokori, Ismaila Ajayi Yusuf, Faith Adedayo Adejumo, Joy Oluwagbohunmi Olatunbosun, Sulaiman Olaide Bukky, Florence Oluwatoyin Akintepede, David B. Olawade, Adetola Emmanuel Babalola, Olamide Asifat, Adeolu Morawo

**Affiliations:** a Department of Medicine and Surgery, Ladoke Akintola University of Technology, Ogbomoso, Nigeria; b Department of Medicine and Surgery, University of Ilorin, Ilorin, Nigeria; c Department of Medicine and Surgery, Obafemi Awolowo University, Ife, Nigeria; d Department of Medicine and Surgery, Bowen University, Iwo, Nigeria; e Department of Medicine, Lagos University Teaching Hospital, Lagos, Nigeria; f Accident and Emergency, Mid Cheshire NHS Trust, Cheshire, UK; g Department of Allied and Public Health, School of Health, Sport and Bioscience, University of East London, London, UK; h Kornberg School of Dentistry, Temple University, Philadelphia, USA; i Department of Medicine, Georgia Southern University, Georgia, USA; j Department of Neurology, Creighton University School of Medicine, Omaha, Nebraska, USA

**Keywords:** cognitive-motor interference, dual-task training (DTT), motor recovery, neuroplasticity, stroke rehabilitation

## Abstract

Stroke is a leading cause of disability and mortality worldwide, with survivors frequently experiencing motor and cognitive impairments that hinder their daily functioning and independence. Dual-task training (DTT), an innovative rehabilitation approach, targets simultaneous improvement in motor and cognitive functions by addressing cognitive-motor interference. This narrative review evaluates the efficacy and safety of DTT in poststroke rehabilitation. PubMed, Google Scholar, Cochrane Library, Scopus, and the Directory of Open Access Journals were searched, yielding 31 studies, including randomized controlled trials and observational studies. Articles were identified that evaluated the safety and efficacy of DTT for poststroke rehabilitation in patients who had suffered from hemorrhagic or ischemic stroke. Before extracting variables, the studies were imported into Rayyan software, and thematic analysis was subsequently conducted using Microsoft Excel. The findings indicate that DTT enhances gait parameters, balance, and cognitive functions, particularly attention and executive function. Additionally, it improves functional outcomes, including activities of daily living, and reduces fall risk. While DTT demonstrates promise in fostering neuroplasticity and improving recovery outcomes, questions regarding its long-term efficacy, optimal implementation, and safety warrant further investigation. This review highlights the potential of DTT as a promising approach in stroke rehabilitation, supporting more integrated recovery strategies.

## Introduction

In adults, stroke stands as the foremost cause of acquired physical disability in middle-to-high income countries^[1]^. According to the World Health Organization, in 2021, stroke was ranked as the third leading cause of death worldwide, following ischemic heart disease and COVID-19^[2]^. It also accounted for a significant portion of disability-adjusted life years, making it the third leading cause in this category as well^[2]^. It impacts motor functions, affecting patients’ mobility, socialization, and even their return to routine activities^[3]^. Reports indicate that 30–70% of stroke survivors experience cognitive impairment, depending on stroke severity and location, comorbidities, and the range of assessment tools used^[4]^. The high prevalence of motor and cognitive impairments highlights the pressing need for more effective rehabilitation strategies. Traditional methods, such as physiotherapy and virtual reality (VR) therapy, face challenges including a lack of patient involvement and slow progress^[5]^. These issues often stem from a focus on isolated tasks and cognitive-motor interference, prompting the exploration of alternative approaches to enhance recovery outcomes for stroke survivors^[6]^.

**HIGHLIGHTS**

- Dual-task training (DTT) significantly improves gait parameters (speed, cadence, stride length), balance (Berg Balance Scale scores), and cognitive functions (attention, executive function, global cognition) in stroke survivors.
- DTT enhances activities of daily living and reduces fall risk, with studies showing improved Functional Independence Measure scores and Activities-specific Balance Confidence scale scores.
- No significant adverse events were reported, highlighting DTT’s safety across supervised settings.

Dual-task training (DTT) has emerged as an innovative approach that not only targets cognitive recovery but also enhances motor functions. This approach is grounded in the principle that cognitive and motor functions are interdependent, and their simultaneous engagement can foster neuroplasticity – the brain’s ability to reorganize itself by forming new neural connections^[7]^. DTT addresses the common challenge of cognitive-motor interference observed in traditional rehabilitation techniques^[8]^. Research has shown that stroke survivors often struggle with dual-task scenarios in daily life, such as walking while talking or carrying an object^[9]^. These challenges are linked to impaired cognitive control and motor coordination, which traditional rehabilitation strategies may not fully address^[10]^. DTT aims to bridge this gap by providing structured training that mimics real-life multitasking demands. This approach not only enhances motor recovery but also improves cognitive domains, such as attention, executive function, and working memory, which are crucial for regaining independence.

The rationale for exploring DTT further lies in its dual benefits for cognitive and motor recovery, its alignment with neurorehabilitation principles, and its promise to address unmet needs in poststroke rehabilitation^[10]^. However, questions remain regarding its optimal implementation, safety, and long-term efficacy, necessitating a thorough review of the existing evidence. This review aims to evaluate the effectiveness and safety of DTT for poststroke rehabilitation. This paper adheres to the TITAN guidelines^[11]^.

## Methods

A narrative review was conducted to explore the efficacy and safety of DTT in poststroke rehabilitation.

### Data sources and search strategy

**Figure 1.**
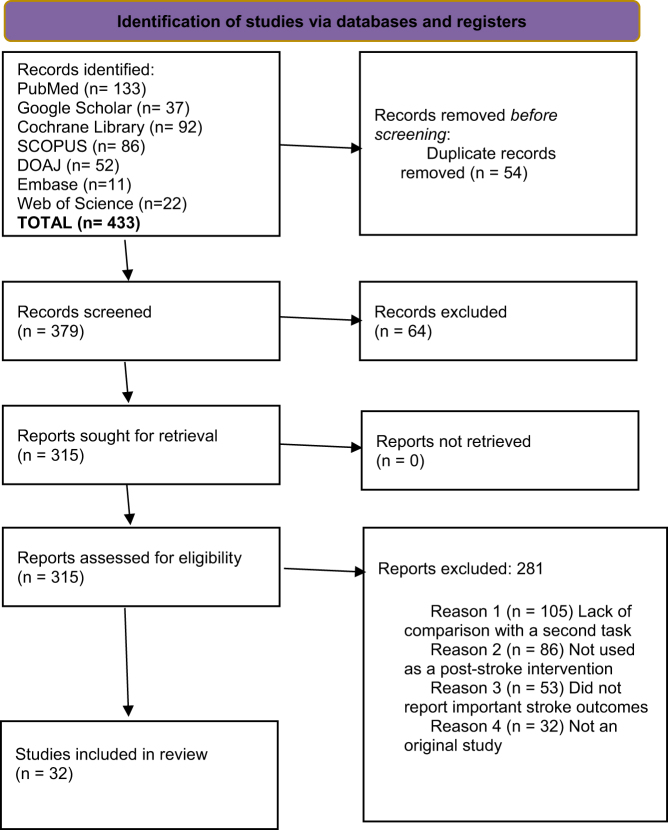
Screening process.

A search was conducted across PubMed/Medline, ScienceDirect, EMBASE, Web of Science, the Cochrane Library, Google Scholar, and the Directory of Open Access Journals. Keywords included “Dual-task training” AND “stroke rehabilitation”), (“Motor-cognitive therapy” AND “stroke rehabilitation”), (“Dual-task training” AND “motor-cognitive therapy” AND “post-stroke rehabilitation”), (“Dual-task training” AND “efficacy” AND “stroke rehabilitation”), (“Dual-task training” AND “safety” AND “post-stroke”), (“Motor-cognitive therapy” AND “efficacy” AND “stroke”), (“Dual-task” AND “post-stroke rehabilitation” AND (“motor-cognitive therapy” OR “cognitive training”)), (“Dual-task training” AND “stroke rehabilitation” AND (“balance” OR “gait”)), and (“Dual-task training” AND “motor-cognitive therapy” AND “stroke recovery”) (Fig. 1). The search spanned from inception to September 2024. Reference lists and grey literature were also screened for additional studies.

### Study eligibility criteria

The inclusion criteria were based on the PICO-S framework:

- *Population*: Adults (≥18 years) diagnosed with ischemic or hemorrhagic stroke.
- *Intervention*: DTT involving simultaneous motor and cognitive or other forms of rehabilitative activities.
- *Comparison*: Conventional single-task rehabilitation or no intervention.
- *Outcomes*: Primary outcomes included motor function (e.g., gait speed, balance) and cognitive performance (e.g., attention, executive function). Secondary outcomes included adherence rates and adverse events.
- *Setting*: Studies conducted in clinical, rehabilitation, or research environments.

#### Inclusion criteria

- Original research articles [e.g., randomized controlled trials (RCTs), cohort studies].
- Human studies.
- Reports with clearly defined intervention protocols and outcome measures.

#### Exclusion criteria

- Non-English studies.

### Data extraction and management

Study files were imported into the Rayyan software, where duplicates were removed. Titles and abstracts were screened independently by two reviewers (IAY and FAA), with discrepancies resolved by consensus with a third reviewer (NA). Extracted data included study design, population characteristics, intervention details, primary and secondary outcomes, and limitations. The final dataset was managed in Microsoft Excel for thematic analysis.

### Data analysis

We conducted a narrative thematic analysis to explore the efficacy and safety of DTT in poststroke rehabilitation. Key themes, including rehabilitation outcomes, training protocols, cognitive-motor integration, and safety considerations, guided the analysis.

### Risk of bias assessment

The quality of included studies was assessed using the Cochrane Risk of Bias tool for RCTs and the Newcastle–Ottawa Scale for observational studies. Two reviewers (IAY, FAA) independently evaluated study quality, with discrepancies resolved by consensus or consultation with a third reviewer (NA). Results were summarized in a supplementary table (Supplemental Digital Content File 1, available at: http://links.lww.com/MS9/B39).

## Results

### Study selection and characteristics

This review analyzed 31 studies^[12–39]^ published between 2007 and 2024 (Table 1). The methodological distribution comprised 16 RCTs, 9 observational or case-control studies, and 7 additional studies using quasi-experimental or pilot designs. The studies exhibited considerable variation in sample size, ranging from small-scale investigations with as few as 2 participants to larger cohorts of up to 104 subjects. The majority of research focused on chronic stroke patients. The demographic profile predominantly included middle-aged to older adults, with participant ages typically ranging from 40 to 65 years. The interventions documented in these studies can be categorized into three primary classifications: cognitive-motor DTT, motor-motor DTT, and technology-enhanced interventions. The studies employed an array of outcome measures across three primary domains: gait parameters, balance and mobility assessments, and cognitive function evaluations. Gait analysis included quantitative measurements of walking speed, stride characteristics (length and time), cadence, step parameters (width and length), single support time, and the dual-task cost on gait performance. Balance and mobility assessments utilized standardized clinical tools such as the Berg Balance Scale (BBS), Timed Up and Go (TUG) Test, and Functional Reach Test (FRT), complemented by instrumental measures of postural sway and center of pressure parameters. Cognitive function assessment included various dimensions, including attention capacity, working memory performance, executive function capabilities, and dual-task interference effects.

**Table 1:** Study characteristics

Authors/year	Study design	Sample size/participant characteristics	Stroke duration (acute/chronic)	Intervention	Comparison	Outcome measures	Efficacy	Safety
Kayabinar *et al*, 2021	Randomized single-blind trial	3	Chronic	VRAGT	VRAGT vs. (RAGT)	10-meter walk, Functional Gait Assessment, Rivermead Mobility Index, BBS, Fall Activity Scale International, and the FIM for gait, mobility, balance, fear of falling, and independence in daily living activities	VR-augmented RAGT improved dual-task gait speeds and dual-task performance of chronic stroke patients; however, there was no difference between the two groups after the treatment	N/A
Pang *et al*, 2018	Single-blinded RCT	84	Chronic	Dual-task balance/mobility training, single-task balance/mobility training, or upper-limb exercise training (control)	Dual-task balance/mobility training vs. single-task balance/mobility training vs. upper limb exercise (control) group	Dual-task interference effectMobility testsSerial-3 subtractions, verbal fluencyFall incidence	Improved dual-task mobility, reducing falls and fall-related injuries in ambulatory chronic stroke patients with intact cognition	N/A
Baetens *et al*, 2013	Prospective cohort study	32	Subacute	Verbal fluency and counting	Single-task gait analysis vs. dual-task gait analysis measured by verbal fluency and counting	FAC, use of walking aid, step length and stride length, fall risk	Decrement of spatial gait characteristics identified fall-prone	N/A
Tisserand *et al*, 2018	Observational study	22	Chronic	Cognitive-motor interference	Single-task (normal gait) vs. four different dual-tasks (cognitive-motor interference)	Margin of stability, base of support, mediolateral dynamic stability, cognitive performance	Poststroke participants presented a larger margin of stability and base of support than controls during single-task	N/A
Kim *et al*, 2021	Double-blinded, pilot RCT	30	Chronic	Dual tDCS with mCIMT	Dual tDCS and mCIMT vs. control group	Fugl-Meyer Assessment scale, MAL	There was a significant improvement in AOU of MAL and usage of unaffected side in the experimental group compared to the control group	N/A
Plummer-D’Amato *et al*, 2007	Observational study	13	Chronic	Cognitive task in combination with walking	Single task (walking or cognitive task alone) vs. dual task (walking + cognitive tasks)	Gait speed, stride time, stride length, cadenceCognitive (reaction time, accuracy and language analysis)	Dual-task effects impacted gait metrics except stride time variability, with speech causing the most interference. Cognitive effects were minimal, limited to speech during walking	N/A
Hollands *et al*, 2014	Observational – case-control	32	Chronic	Single task, dual motor and dual cognitive	Effects of dual tasks on turning ability in stroke patients vs. age-matched controls	Time to turn, variability in time to turn, step length, step width, single support time	Both groups took longer, were more variable, tended to widen the second step and, crucially, increased single support time on the inside leg of the turn while turning and distracted	N/A
Rogalski *et al*, 2010	Observational study	13	Chronic	Talking and walking	Single task (talking) vs. dual task (talking and walking)	MMSE, Stroop, WASI Vocabulary	Although there were no effects of the dual task on coherence, global coherence was significantly disrupted regardless of the single- or dual-task condition	N/A
Melzer *et al*, 2010	Observational	10	Chronic	Voluntary step execution test	Single-task vs. dual-task conditions in stroke survivors vs. controls	Step time, step initiation phase, swing phase, push-off power during stepping	For dual compared to single task, the stepping time increased significantly due to a significant increase in the duration of step initiation	N/A
Yang *et al*, 2007	Single-blind RCT	25	Chronic	4 weeks ball exercise program	Experimental group vs. control group	Walking speed, cadence, stride time, stride length, temporal symmetry index	The dual-task-based exercise program is feasible and beneficial for improving walking ability in subjects with chronic stroke	N/A
Melzer *et al*, 2009	Case-control study	32	Chronic	Forward and backward rapid voluntary stepping	Chronic stroke survivors vs. healthy controls	Voluntary step behavior, step initiation, preparatory and swing phases, foot-off time, foot-contact time	Chronic stroke survivors showed slower step parameters than controls in all tasks. Dual-tasking increased foot-contact time by 12% in stroke survivors and 15% in controls	N/A
Fishbein *et al*, 2019	RCT	22	Chronic	Virtual reality-based DTW and single-task TMW	DTW vs. TMW	10-meter walking test, TUG, FRT, LRT, ABC scale, BBS	The DTW group showed significant improvements in balance (BBS, FRT, LRT-L/R, *P* < 0.01), gait (10 mW time, *P* < 0.05), and ABC scale (*P* < 0.01), with no interaction changes in TUG	N/A
Aneksan *et al*, 2022	RCT	25	Subacute	dual-tDCS combined with task-specific training	Active dual-tDCS group vs. sham dual-tDCS group	Gait speed, temporospatial gait variables, lower-limb functional tasks, muscle strength	Both groups showed improvement in primary and secondary outcomes, with no significant differences and small to moderate effect sizes for active vs. sham tDCS	N/A
Bensoussan *et al*, 2007	Retrospective study	46	Chronic	EO-AT	Hemiplegic patients vs. healthy controls	Sway area and sway path	In hemiplegic patients, body sway area increased with EC (*P* < 0.001) and EO-AT (*P* < 0.017) compared to EO but was smaller with EO-AT than EC (*P* < 0.014). Healthy subjects showed no significant differences between EO-AT and EO (*P* < 0.42). In hemiplegic patients, sway area and path during EO-AT correlated with age	N/A
Ayodogdu *et al*, 2019	RCT	53	Chronic	DTT while walking	Intervention group vs. control group	Balance, mobility, functional independence, fear of falling	Both groups showed significant improvements in all parameters posttreatment (*P* < 0.05)	N/A
Plummer *et al*, 2020	RCT	3	Chronic	DTGT	DTGT vs. STGT	Relative DTEg, cognitive task performance (DTEc)	No treatment effects were found on DTEg or DTEc in either group at any walking speed. All participants showed improved single and dual-task gait speeds without changes in the dual-task effect	N/A
Park *et al*, 2019	RCT	53	Chronic	DTT while walking	Intervention group vs. control group	Balance, mobility, functional independence, fear of falling	Post-intervention, the dual-task group outperformed the occupational therapy group in DST-Forward (*P* = 0.04), DST-Backward (*P* = 0.001), ST-Color (*P* = 0.023), and BBS (*P* = 0.009)	N/A
Liu *et al*, 2017	Randomized controlled pilot trial	28	Chronic	CDTT, MDTT, or CPT	CDTT group vs. MDTT group vs. CPT group	Gait speed, dual-task cost of gait speed (DTC-speed), cadence, stride time, stride length	After CDTT, cognitive-motor dual-task gait performance (stride length, DTC-speed) improved (*P* = 0.021, *P* = 0.015). After MDTT, motor dual-task gait performance (gait speed, stride length, DTC-speed) improved (*P* = 0.008 for all)	N/A
Maeneja *et al*, 2023	RCT	34	Subacute	Aerobic physical exercise (PE) group vs. dual-task (DT) gait exercise group	PE vs. DE group	Mini-Mental State Examination (MMSE), d2 Test of Attention, Visual Analog Scale, Borg Scale of perceived exertion	A mixed model ANOVA revealed a significant interaction effect with a large effect size for most cognitive variables. Significant group differences were observed for the d2 Test of Attention, primarily from T0 to T2. ANOVA for MMSE also showed a significant interaction effect with improvements from T0 to T2	N/A
Chan and Tsang, 2017	Comparative study	104	Chronic	Motor tests	Stroke survivors vs. non-stroke controls, single-tasking vs. dual-tasking	Auditory Stroop test (reaction time, accuracy), turning-while-walking test (turning duration, number of steps, completion time)	Stroke survivors had reduced accuracy in the auditory Stroop test during dual-tasking, with no change in reaction time. They also took longer and needed more steps for the turning-while-walking task, performing worse than controls in both conditions	N/A
Schinkel-Ivy *et al*, 2016	Experimental	42	Subacute	Reactive stepping trials with and without a secondary cognitive task (dual-task condition)	Usual-response (single-task) trials vs. dual-task trials, first trial vs. subsequent trials	Step timing, number of steps, time of unloading onset	The first usual-response trial had a longer unloading onset and more steps than later trials. No significant differences were found between usual-response and dual-task trials. Performance improved in usual-response trials but declined with the dual-task condition	N/A
Sun *et al*, 2022	RCT	40	Chronic	CMDT training vs. cognitive task (CT) training	CMDT vs. CT group	Mini-mental State Examination (MMSE), MoCA, event-related potentials (ERP), functional near-infrared spectroscopy (fNIRS)	The CMDT group showed significant improvements in MMSE (*P* = 0.01) and MoCA (*P* = 0.024) compared to the CT group. ERP and fNIRS results indicated CMDT shortened P300 latency (*P* = 0.001) and oxygenated hemoglobin peak time (*P* = 0.004)	N/A
Jiejiao *et al*, 2012	RCT	100	Chronic	Cognitive DTT (conventional balance program + cognitive training) vs. conventional balance program (single-task training)	Training group vs. control group	Static postural control, center of pressure sway, maximum displacement, anteroposterior balance indices	The control group had significantly greater medial-lateral displacement than the training group (*P* < 0.05). The dual-task group showed significantly better anteroposterior balance with EO after 8 weeks (*P* = 0.000)	N/A
Kim *et al*, 2019	RCT	25	Chronic	DTT consisting of obstacle/non-obstacle walking + cognitive tasks + general physical therapy	Obstacle walking group vs. non-obstacle walking group	FRT for balance, Gait Analyzer (G-Walk) for gait cadence, velocity, and stride length, FIM for functional assessment	DTT with cognitive tasks and walking improved balance, gait, and function in stroke patients, with obstacle walking training more effective than non-obstacle training for ADLs	N/A
YIN *et al*, 2023	Observational	40	Chronic	Dual-task paradigms (motor-motor and cognitive-motor DTW)	Single-task walking vs. motor-motor DTW vs. cognitive-motor DTW	Gait speed, Step frequency, Step size, Proportion of double support phase time, Step duration, Trunk coronal plane and sagittal plane swing angles	Motor-motor DTW decreased trunk swing angles, while cognitive-motor DTW increased gait speed, double support phase time, and trunk swing angle. Cognitive-motor DTW showed greater dual-task costs in gait speed, step size, and double support phase time (*P* < 0.05)	N/A
Shim *et al*, 2012	RCT	33	Motor task	MDTT (30 minutes, 3 days/week, 6 weeks) + physical therapy (30 minutes, 5 days/week, 6 weeks)	MDTT group vs. control group (physical therapy only)	Temporal parameters (gait speed, cadence), spatial parameters (paretic/non-paretic step/stride length, single limb support period)	MDTT significantly improved gait speed, cadence, step length, stride length, and single limb support, with notable differences between the training and control groups	N/A
Choi *et al*, 2024	RCRCT	40	Chronic	Dual-task exercise intervention (30 minutes, 3 times/week, 4 weeks)	Dual-task exercise intervention group vs. single exercise intervention group	Lung function, balance, ADLs	Significant improvements in lung function, balance, and ADLs were found only in the experimental group (*P* < 0.01). Between-group comparisons also showed significant improvements (*P* < 0.05 for lung function and balance, *P* < 0.01 for ADLs)	N/A
Cho and Lee, 2010	RCT	25	Chronic	Physical therapy (5 sessions/week, 30 minutes/session, 6 weeks) + Two MDTT (30 minutes/day, 3 days/week, 6 weeks)	Experimental group vs. control group	Static balance (postural sway area with open/closed eyes on foam surface), Dynamic balance, Gait function	The group that practiced the additional two MDTT showed significant improvements in postural sway area (open and closed eyes on foam surface), dynamic balance (*P* < 0.05), and gait function (*P* < 0.05).	N/A
Park *et al*, 2011	RCT	25	Chronic	DTT + General Physical Therapy (50 minutes/day, 3 days/week, 6 weeks) + General Physical Therapy (30 minutes/day, 5 days/week, 6 weeks)	DTT group vs. control group (General Physical Therapy only)	TIS, PASS, Postural sway (eye opened and eye closed) on Force Plate	Postural stability and balance significantly improved after training in the DTT group (*P* < 0.05)	N/A
Poon, 2017	Interventional study	2	Chronic	DTT	Pre-intervention vs. post-intervention	Hand function: Box and Block Test, Balance ability: BBS	Significant improvements in hand function and balance ability after DTT	N/A
Walshe *et al*, 2019	Cross-sectional study, pilot study	24	Chronic	Working memory (2-back) and inhibition (Stroop) tasks	Chronic stroke survivors vs. healthy controls, executive tasks (2-back, Stroop) vs. non-executive task (motor response)	Gait speed, stride time, stride time variability, stride length, stride length variability, cognitive task performance (accuracy)	Stroke survivors had slower gait speeds across conditions, but no significant group differences were found in spatial or temporal gait characteristics. Gait performance was maintained during executive and non-executive dual tasks, with no significant effect of group on cognitive task performance (all *P* > 0.052)	N/A
Bang *et al*, 2012	RCT	14	Chronic	Treadmill training + cognitive task (4 weeks)	Experimental group vs. control group	10-meter walking test (walking speed), TUG test (dynamic balance), 6-minutes walking (6-m walking) test (walking endurance)	Both groups showed significant improvements in the 10 m (*P* 0.05). TUG test improvements were significant for both groups (*P* < 0.001) and between groups (*P* < 0.01)	

ABC scale, Activities-specific Balance Confidence scale; ADLs, activities of daily living; AOU, amount of use; BBS, Berg Balance Scale; CDTT, cognitive dual-task gait training; CPT, conventional physical therapy; CMDT, cognitive-motor dual-task; DTEg, dual-task effect on gait speed; DTGT, dual-task gait training; DTW, dual-task walking; DTT, dual-task training, dual-tDCS, dual-transcranial direct current stimulation; EO, eyes open; EO-AT, eyes open with arithmetic task; FIM, Functional Independence Measure; FRT, Functional Reach Test; LRT, Lateral Reach Test; MAL, Motor Activity Log; mCIMT, modified constraint-induced movement therapy; MDTT, motor dual-task gait training; MoCA, Montreal Cognitive Assessment; PASS, Postural Assessment Scale for Stroke; RCT, randomized controlled trial; STGT, single-task gait training; TIS, Trunk Impairment Scale; TMW, treadmill walking; TUG, Timed Up and Go; VRAGT, virtual reality augmented gait training.

### Motor outcomes

## Gait and mobility

DTT has been shown to significantly improve gait parameters across multiple studies. RCTs reported enhanced gait speed^[26,33,39]^, cadence^[20,32,33]^, and stride length^[33,40]^. Yang *et al* (2007) found that a ball exercise DTT program improved walking speed and cadence (*P* < 0.05)^[20]^. Shim *et al* (2012) reported significant improvements in gait speed, step length, and cadence with motor DTT (*P* < 0.05)^[33]^. Bang *et al* (2012) noted enhanced 10-meter walk test performance (*P* < 0.01)^[39]^.

VR-based DTT also enhanced gait. Fishbein *et al* (2019) noted significant improvements in 10-meter walk test times (*P* < 0.05)^[22]^, while Kayabinar *et al* (2021) found VR-augmented gait training improved dual-task gait speeds, though not significantly better than robot-assisted gait training^[12]^. Plummer *et al* (2020) observed improved single- and dual-task gait speeds in a small RCT (*n* = 3), but no change in dual-task effect on gait^[26]^.

Mobility outcomes, such as TUG test performance, showed mixed results. Bang *et al* (2012) reported significant TUG improvements in both DTT and control groups (*P* < 0.001), with between-group differences favoring DTT (*P* < 0.01)^[39]^. However, Fishbein *et al* (2019) and Plummer *et al* (2020) found no significant changes in TUG, possibly due to small sample sizes or task complexity^[22,26]^.

## Balance

Balance improvements were consistently reported, with DTT enhancing BBS scores in multiple RCTs^[12,22,34]^. Fishbein *et al* (2019) reported significant BBS improvements in VR-based DTT (*P* < 0.01), alongside FRT and Lateral Reach Test gains (*P* < 0.01)^[22]^. Jiejiao *et al* (2012) demonstrated improved anteroposterior balance (*P* = 0.000) and reduced medial-lateral sway in cognitive DTT (*P* < 0.05)^[31]^. Cho and Lee (2010) and Park *et al* (2011) reported reduced postural sway area (*P* < 0.05), enhancing dynamic and static balance^[35,36]^. Obstacle-based DTT further improved balance. Kim *et al* (2019) found significant improvements in FRT during obstacle walking DTT compared to non-obstacle training^[32]^. Choi *et al* (2024) noted significant balance gains in DTT vs. single-task exercise (*P* < 0.05)^[34]^.

## Upper limb function

Evidence for upper limb function was limited but promising. Kim *et al* (2021) conducted a double-blind RCT (*n* = 30, chronic, mean age 55) combining dual-transcranial direct current stimulation (tDCS) with modified constraint-induced movement therapy (30 min, 5×/week, 2 weeks), reporting significant improvements in motor activity log amount of use scores (*P* < 0.05) and increased use of the unaffected side compared to sham tDCS^[15]^.

### Cognitive outcomes

DTT improved cognitive function, particularly in attention, executive function, and global cognition. Sun *et al* (2022) reported significant improvements in Mini-Mental State Examination (MMSE) scores (*P* = 0.01) and Montreal Cognitive Assessment scores (*P* = 0.024) in cognitive-motor DTT compared to cognitive-only training^[30]^. Maeneja *et al* (2023) found significant interaction effects for MMSE and d2 Test of Attention (*P* < 0.05), with DTT outperforming aerobic exercise from baseline to follow-up^[27]^. Chan and Tsang (2017) noted reduced accuracy in the Auditory Stroop test during dual-tasking (*P* < 0.05), indicating cognitive challenge but improved performance post-training^[28]^. Neurophysiological markers supported cognitive gains. Sun *et al* (2022) found that cognitive-motor DTT shortened P300 latency (*P* = 0.001) and oxygenated hemoglobin peak time (*P* = 0.004), indicating enhanced neural reactivity^[30]^. Plummer-D’Amato *et al* (2007) reported minimal cognitive interference except for speech tasks, which significantly impacted gait (*P* < 0.05)^[16]^. Pang *et al* (2018) observed improved verbal fluency and serial-3 subtraction accuracy under dual-task conditions, reducing cognitive-motor interference^[13]^.

## Functional independence

DTT enhanced functional independence, particularly in activities of daily living (ADLs). Kim *et al* (2019) reported improved Functional Independence Measure (FIM) scores in obstacle-based DTT, with greater benefits in ADLs than non-obstacle training (*P* < 0.05)^[32]^. Choi *et al* (2024) found significant improvements in ADLs in DTT compared to single-task exercise (*P* < 0.01)^[34]^. Kayabinar *et al* (2021) noted enhanced FIM scores in VR-based DTT, although not significantly better than those of the controls^[12]^. Aydogdu *et al* (2019) reported improved mobility and functional independence (*P* < 0.05)^[25]^.

## Fall risk

DTT reduced the risk of falls and fear of falling. Pang *et al* (2018) reported a decrease in fall incidence among chronic stroke survivors with intact cognition, along with improved dual-task mobility^[13]^. Aydogdu *et al* (2019) noted reduced fear of falling (*P* < 0.05), linked to enhanced balance and mobility^[25]^. Baetens *et al* (2013) identified fall-prone patients via gait decrement under dual-task conditions, suggesting that DTT has diagnostic utility^[40]^. Fishbein *et al* (2019) reported improved Activities-specific Balance Confidence scale scores (*P* < 0.01), reinforcing the impact of DTT on fall prevention^[22]^.

### Safety

No studies reported significant adverse events (e.g., falls, injuries) during DTT, indicating a favorable safety profile^[12–40]^. Interventions were supervised, with tasks tailored to participants’ cognitive and motor abilities, ensuring feasibility across chronic and subacute stroke settings. Studies by Pang *et al* (2018) and Aydogdu *et al* (2019) noted a reduced incidence of falls, further supporting the safety of DTT for community ambulation^[13,25]^.

### Comparative efficacy

DTT generally outperformed single-task training, conventional therapy, or sham interventions. Jiejiao *et al* (2012) reported better balance and mobility in the DTT groups compared to controls (*P* < 0.05)^[31]^. Choi *et al* (2024) noted significant improvements in balance and ADLs with DTT compared to single-task exercise (*P* < 0.01)^[34]^. However, Kayabinar *et al* (2021) found no significant difference between VR-based DTT and robot-assisted gait training^[12]^, and Aneksan *et al* (2022) reported no advantage of dual-tDCS over sham tDCS, possibly due to small effect sizes or sample limitations^[23]^.

### Limitations and considerations

Several methodological limitations were noted across the reviewed studies. Many studies had small sample sizes, which reduced the generalizability of the findings. For instance, Kayabinar *et al* (2021) and Yang *et al* (2007) relied on limited participant numbers, which could affect the robustness of their conclusions. Additionally, the studies employed varied intervention protocols, including different types of DTT (e.g., VR-based, balance-focused, or cognitive-motor integration tasks), making it challenging to establish standardized guidelines. Outcome measures also varied significantly, with studies using tools such as the BBS, 10-meter walk test, or dual-task cost, complicating direct comparisons between interventions.

The majority of studies focused on chronic stroke patients, leaving a gap in evidence for individuals in the acute or subacute phases of recovery. Furthermore, participants in the studies demonstrated varying levels of initial impairment, ranging from mild to severe motor and cognitive deficits, which could influence the effectiveness of DTT. This heterogeneity highlights the need for more targeted studies in specific subgroups of stroke patients.

Our review is not without limitations. A significant limitation was the inclusion of only studies published in the English language. The decision to include only English-language studies was made due to resource constraints. Future research should explore multilingual studies to provide a more comprehensive understanding of the topic. Limited evidence on long-term outcomes (>6 months) and cost-effectiveness of DTT, particularly for resource-intensive interventions like VR or robotics, restricted conclusions about sustained benefits and scalability.

## Clinical implications

The integration of DTT into stroke rehabilitation holds significant promise, yet its clinical application requires a well-structured approach. A key consideration is the principle of progressive difficulty, which ensures that tasks remain challenging but manageable for patients. Initial training may involve simpler combinations of motor and cognitive tasks, such as walking while reciting numbers, which can gradually progress to more complex exercises, such as navigating obstacles while engaging in memory recall. This progression minimizes cognitive and physical overload, fostering sustained engagement and gradual improvement in both domains.

An individualized approach to DTT is equally essential. Variations in stroke severity, stages of recovery (acute, subacute, or chronic), and patient-specific rehabilitation goals necessitate personalised interventions. Tailoring protocols to each patient’s baseline capabilities ensures that both motor and cognitive improvements are effectively targeted. Regular assessments of motor and cognitive performance further enable clinicians to dynamically adjust intervention strategies, maintaining the appropriate level of challenge throughout the rehabilitation process.

Safety remains a critical concern in the implementation of DTT. Proper pre-assessments of cognitive and motor function are necessary to determine a patient’s readiness for dual-task exercises, as not all individuals may initially possess the capacity to manage concurrent task demands. For patients with significant impairments, preparatory single-task training may serve as a foundation before progressing to dual-task protocols.

The gradual escalation of task difficulty is crucial to minimizing risks such as falls, frustration, or physical and mental exhaustion. Clinicians must monitor patients for signs of fatigue, such as reduced task accuracy or slowed motor responses, which may indicate the need for adjustments in task intensity or frequency. Additionally, implementing robust safety measures, such as harnesses during balance training or closely supervised sessions, can further mitigate risks, ensuring that patients benefit from DTT in a controlled environment.

Despite its promise, the accessibility of DTT remains a significant challenge, particularly in resource-limited settings. The high costs associated with advanced technologies, such as VR and robotics, often place these tools out of reach for many healthcare facilities and patients. Additionally, the availability of trained therapists proficient in using these technologies is limited, further restricting their implementation. To bridge these gaps, innovative strategies are needed. Developing low-cost or portable DTT devices could make these interventions more accessible to underserved populations. Tele-rehabilitation programs incorporating DTT elements offer another viable solution, enabling remote therapy delivery to patients in geographically isolated areas. Furthermore, open-source software for VR or AI-based tools could democratize access to cutting-edge rehabilitation technologies. Inclusive policies and dedicated funding initiatives are critical to addressing these disparities. Governments and healthcare organizations must prioritize investments in DTT infrastructure and training programs to ensure that these innovative approaches are equitably distributed.

## Conclusion

DTT represents a promising approach in stroke rehabilitation, addressing the dual challenges of motor and cognitive impairments that traditional single-task approaches often fail to resolve. This review highlights its efficacy in improving gait, balance, cognitive performance, and functional outcomes, including fall risk and ADLs. DTT offers an integrated approach that mirrors real-world multitasking demands, equipping stroke survivors with the skills necessary for regaining independence. Despite these encouraging findings, the implementation of DTT in clinical practice requires careful consideration and evaluation. Variability in training protocols, intervention durations, and participant characteristics across studies complicates the establishment of standardized guidelines. Future research should focus on large-scale, multicenter trials to determine the optimal parameters for DTT, explore its long-term benefits, and assess its cost-effectiveness in diverse healthcare settings.

## Data Availability

Data sharing is not applicable to this article as there were no datasets.
